# Depression, Anxiety and Quality of Life among Online Responders in Poland: A Cross-Sectional Study Covering Four Waves of the COVID-19 Pandemic

**DOI:** 10.3390/ijerph19169934

**Published:** 2022-08-11

**Authors:** Mateusz Babicki, Bogna Bogudzińska, Krzysztof Kowalski, Agnieszka Mastalerz-Migas

**Affiliations:** 1 Department of Family Medicine, Wroclaw Medical University, 51-141 Wroclaw, Poland; 2 Faculty of Medicine, Wroclaw Medical University, 50-367 Wroclaw, Poland; 3 Department of Psychiatry, Division of Consultation Psychiatry and Neuroscience, Wroclaw Medical University, 50-367 Wroclaw, Poland

**Keywords:** anxiety, depression, COVID-19, mental health, quality of life

## Abstract

**Introduction:** The COVID-19 pandemic has affected the mental health of the population. This study aims to assess the prevalence of subjective depressive and anxiety symptoms as well as assess the quality of life in different waves of the COVID-19 pandemic based on an online survey. **Methods:** The study was conducted based on an original and anonymous questionnaire, consisting of a section assessing sociodemographic status and psychometric tools: Beck Depression Inventory (BDI), Generalised Anxiety Disorder Assessment (GAD-7) and Manchester Short Assessment of Quality of Life (MANSA). A total of 6739 people participated in the survey, with the largest number from the first wave of the pandemic (2467—36.6%), followed by 1627 (24.1%) for the second wave, 1696 (25.2%) for wave three and 949 (14.1%) for wave four. The mean age of the study group was 28.19 ± 9.94. **Results:** There was an initial, gradual increase in depressive and anxiety symptoms over the three waves. There were no significant differences in the quality-of-life scores, except for the second and third waves (−0.0846; *p* = 0.013. It was found that women, residents of big cities and people with psychiatric history showed higher BDI and GAD-7 scores. **Conclusions:** The impact of the pandemic on mental health was not homogeneous, with the first three waves of the COVID-19 pandemic having more of an impact compared to the fourth wave. Female respondents’ sex, history of mental disease and reduced earning capacity exacerbated psychiatric symptoms.

## 1. Introduction

It has already been 2 years since the World Health Organisation (WHO) declared the pandemic. It has left its mark on many aspects of human life, including mental health [1]. In Poland, as well as in many other European countries, four waves of infections have been distinguished so far, together with the fifth one which was caused by the Omicron variant (VOC- variant of concern) [2,3,4]. The fourth wave, associated with VOC delta, which was the cause of 99% of infections in Poland at the time, was characterised by accelerated transmission compared to the previous three waves [5,6]. This variant was also found to be more dangerous for younger people, which resulted in a higher risk of hospitalisations, including ICU admissions, in this age group (especially in 30–59 years old subgroup according to meta-analysis) [7]. Additionally, the efficacy of the vaccines was lower than in the initial observations due to the antigenic difference [8,9,10]. Moreover, many people in Poland were still unvaccinated (55.6% of the population was vaccinated by the end of 2021 compared to the EU average of 68.5%), which, together with an overburdened health system, might have contributed to the high number of deaths [6]. There was also a lack of effective outpatient causal treatment for COVID-19 during this period. All of this might have had an impact on people’s mental well-being, as escalated preoccupation with one’s own life and health negatively affects mental well-being, especially in young people. It was found that in this age group, concern about COVID-19 was positively correlated with anxiety levels, which was not the case in people aged over 50 [11]. However, in older adults, the COVID-19 pandemic impacted negatively on the course of dementia and other neuropsychiatric disorders due to social isolation and difficult access to treatment [12].

Data from a meta-analysis suggest that depressive and anxiety symptoms increased during the pandemic [13]. Socio-demographic factors were found to play a crucial role in the intensity of these symptoms. The elderly and men coped better with stress [14,15]. Anxiety about the sense of one’s autonomy and the quality of social relationships have been shown to contribute to increased anxiety and depression symptoms among Polish young adults. In contrast, older people were less anxious about job loss [16]. Women were perceived to be more stress-sensitive and, hence, more likely to suffer from anxiety, depression and sleep disorders during lockdowns [17]. Furthermore, social distancing restrictions intensified the feeling of loneliness, which led to increased alcohol consumption, excessive consumption of which promotes severe COVID-19 as well as depression [18,19,20]. At the same time, restrictions made it difficult to practice many sports. Meanwhile, physical activity is considered a strong protector of mental health [21]. Such limitations resulted in a significant decline in scores on a scale of subjectively perceived quality of life compared to pre-pandemic times, which, in addition to aspects directly related to mental health, also included aspects such as sexual satisfaction and quality of social relationships [22].

Interestingly, some studies have shown that the mental resilience of the population increases along with the duration of a stressful situation [23]. The relationship between attitudes towards vaccination and Poles’ mental health seems particularly relevant [22]. Fully vaccinated people reported lower levels of anxiety and a higher quality of life than those awaiting vaccination. On the other hand, vaccine deniers downplayed the threat and showed the lowest level of anxiety. This may be because they are mainly people with extreme views who deny both the sense of vaccination and the existence of the pandemic as a whole [22].

During the first wave (March 2020–May 2020) of the pandemic, there were radical restrictions in Poland. The entire education sector was closed, as well as nurseries and kindergartens. The gastronomy, hotel industry, fitness industry as well as the small service sector were closed. In addition, access to recreational areas, including forests, was blocked. However, during the second wave (II wave: October 2020), the country was divided into regions and different, real-time levels of restrictions due to the number of infections. During the third wave (February 2021–May 2021), specific exceptions were introduced, e.g., for licensed athletes—gyms, and for children of health care workers—kindergartens [24]. In the course of the fourth wave (October 2021–December 2021), restrictions in Poland were more lenient than before, limited to social distancing and wearing masks. Additionally, they were poorly respected compared to the previous waves, including fewer people wearing masks in public places or complying with occupancy limits [25]. The worsening of economic situation, especially the rise in inflation (prices of goods in Poland increased by 7.8% compared to November 2021, the month one year prior to the peak of fourth wave), which has been confirmed by previous studies, and which also results from the ongoing epidemiological situation, may also aggravate anxiety [26,27]. Another negative factor is that a significant part of society are not aware of effective techniques for coping with stress [28].

To our knowledge, no study has yet been published assessing psychological well-being in terms of depression and anxiety symptoms and the quality of life of Poles throughout all four waves of the pandemic. This study aimed to fill this gap, focusing on the last wave due to its distinctive character. The following hypotheses were made:

1. Following adaptation to the situation and more lenient restrictions, the intensity of anxiety and depressive disorders during the fourth wave of infections was less severe than in the previous pandemic waves;
2. Women and youth were more prone to mental disorders during the pandemic;
3. The economic situation is an important predictor of mental health during the pandemic.

## 2. Materials and Methods

### 2.1. Methods

The present study was based on an online questionnaire (Google forms) and distributed via social media (Facebook’s groups on various topics, both COVID-19 and general forums). It was addressed to Polish residents aged 18 and over with Internet access. Participation in the study was voluntary and fully anonymous. The participants were free to withdraw from the study at any stage without giving a reason. Before they participated in the study, the respondents were informed in preliminary section of the Google forms about the nature of the study, its methodology and objectives and, after that, informed consent was obtained from those willing to participate. Respondents who provided their age in the questionnaire under 18 were excluded from the study.

The study comprised four stages, corresponding to particular waves of increase in COVID-19 cases in Poland. The first stage covered the period from 17 to 26 April 2020, when the incidence rates were the lowest during the past 2 years, ranging from 263 to 460 cases of COVID-19, with a daily death rate of 18–40 [29]. As this was the first period of the pandemic, knowledge of the virus and how to control it was scarce; the government introduced several restrictions, including the closure of schools, shops (excluding food stores), cultural centres, the cosmetics industry and hotels [28]. The study was repeated during another increase in COVID-19 cases in Poland, and the questionnaire was distributed in the period from 1 to 30 December 2020, when the daily incidence ranged from 2921 to 14,835 cases and there were 29–620 deaths per day [30]. After a short period without restrictions, some restrictions were reinstated, excluding the beauty industry and shopping malls [31]. For the third stage, data were collected from 20 March 2021 to 30 April 2021, when the daily incidence ranged from 6802 to 35,246 cases of COVID-19, with daily deaths ranging from 428 to 954 [29]. These increases have led to a tightening of existing restrictions, which involved the closure of educational establishments, nurseries and kindergartens, shopping malls, construction shops and cultural establishments. There was also a considerable emphasis on the possibility of remote working [32]. The final monitoring stage from 1 to 31 November 2021 coincided with the wave with the highest incidence and mortality rates to date, ranging from 9839 to 29,062 COVID-19 cases and 209 to 793 deaths [29]. Shops and malls were not closed during this wave of infections, and up to 75% occupancy limits were introduced in sports venues, cinemas and restaurants. Additionally, remote learning was introduced for primary and secondary school students [33]. This was the period with the most lenient restrictions in Poland to date.

The present study was approved by the Bioethics Committee of the Wroclaw Medical University (approval number: KB-471/2020) and was conducted in accordance with the Declaration of Helsinki.

The study used an original questionnaire consisting of several parts. It included single-choice and closed-ended questions. The first part included the questions concerning sociodemographic data such as age, gender, education level or relationship status. The fact of being a health care worker was also verified together with the psychiatric history. In the next section, authors’ questions were used to assess the level of anxiety about COVID-19. The first was to assess the anxiety of COVID-19 compared to other illnesses, with the following responses to choose from: “No, I am not afraid/I am less afraid of other diseases/I am as afraid as other diseases/I am more afraid than other diseases”. In addition, the questions based on a 10-point Likert scale, where 1 is no anxiety and 10 is extreme anxiety, were used to assess anxiety in relation to COVID-19, neighbour quarantine and neighbour disease. This part of the questionnaire also verified the respondents’ attitudes towards compliance with the measures taken by the government to stem the spread of COVID-19.

The final stage of the study consisted of standardised psychometric tools such as the Beck Depression Inventory (BDI), the Generalised Anxiety Disorder Assessment (GAD-7) and the Manchester Short Assessment of Quality of Life (MANSA). All of them have been validated on various populations as scales of high sensitivity and specificity [34,35,36]. On the basis of our own research, the reliability of the scales was estimated within the Alfa Cronbach. The values of 0.917 for BDI, 0.921 for GAD-7 and 0.849 for MANSA were obtained.

1. Beck Depression Inventory (BDI) is a commonly used tool to measure depression. It consists of 21 questions, with responses on a 0–3 scale. Interpretation of the tool is based on the summed point value where the following cut-off points were applied: 0–11—no depression, 12–26 points—mild depression; 27–49—moderate depression; and 50–63—severe depression [37,38];
2. Generalised Anxiety Disorder Assessment (GAD-7) is a psychometric tool for assessing anxiety. The tool consists of 7 questions based on a 4-grade Likert scale, where respondents rate the frequency of occurrence of a given psychological state over the past 14 days (0—does not occur, 1—a few days, 2—more than half the time, 3—almost always). The analysis is based on summed scores, with cut-off points of 5, 10 and 15 indicating the presence of mild, moderate and severe anxiety, respectively [39];
3. Manchester Short Assessment of Quality of Life (MANSA) is a commonly used psychometric tool for the subjective assessment of the quality of life by evaluating 16 aspects of life. When creating the survey, questions based on a 7-point Likert scale were used (1—could not be worse, 7—could not be better). The higher the total score, the higher the quality of life is assessed [40,41].

### 2.2. Participants

A total of 6739 respondents took part in the study in four stages. Participants in the first stage of the study were the most numerous group—2467 respondents. At each stage, women, people having a university education and living in a city of more than 250,000 inhabitants were in the clear majority. The mean age of the study group was 28.19 ± 9.94.

### 2.3. Statistical Analysis

In the present study, variables were of qualitative and quantitative nature. The analysis of quantitative variables was based on the use of basic descriptive statistics. The Lilliefors test was used to assess the normality of the distribution and the Brown–Forsythe test to assess variance. If the assumption of equal variances was not met, a Welch’s ANOVA was performed with an in-depth post hoc analysis using the Games-Howell test. Pearson’s chi-squared test with Bonferroni correction was used to compare sociodemographic variables of qualitative nature.

The effect of sociodemographic variables on individual scales was assessed using linear models. In each case, a statistical significance level of <0.05 was assumed. Statistical analysis was performed using Statistica 14.0.0.15 software (TIBCO Software, Palo Alto, CA, USA).

## 3. Results

### 3.1. Characteristics of the Study Group

There were significant differences in mean age of respondents comprising 32.2 ± 10.72; 24.6 ± 7.06; 27.83 ± 9.55; 29.7 ± 9.60 for stages 1, 2, 3 and 4 of the study, respectively. No statistically significant differences were found between participants at each wave in the percentage of previous users of psychiatric services and those receiving psychiatric medication. In addition, the percentage of people who lost their source of income due to the pandemic was lowest in the fourth wave of the pandemic. A detailed comparative breakdown of participants in all pandemic waves is presented in Table 1.

### 3.2. Analysis of BDI, GAD-7 and MANSA for Each Wave of the COVID-19 Pandemic

**Figure 1:**
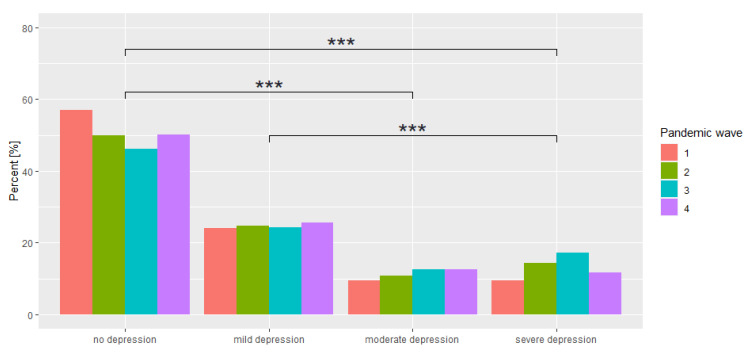
Comparison of BDI interpretations across waves of the study. *** *p* < 0.001.

Analysis of mean BDI scores revealed that as the COVID-19 pandemic continued, there was an increase up to wave 3, with the greatest difference shown between waves 1 and 3 *p* < 0.001. There was a subsequent decrease in mean values of 0.0578 points between waves 3 and 4 (*p* = 0.027). In the BDI interpretation, it can be observed that there is no statistically significant difference between adjacent interpretations (no depression—mild depression (*p* = 0.087), mild depression—moderate depression (*p* = 0.327), moderate depression—severe depression (*p* = 0.144), while a statistically significant difference was observed for pairs no depression—moderate depression (*p* < 0.001), no depression—severe depression (*p* < 0.001) and mild depression—severe depression (*p* < 0.001) for all study stages. A comparison is presented in Figure 1.

**Figure 2:**
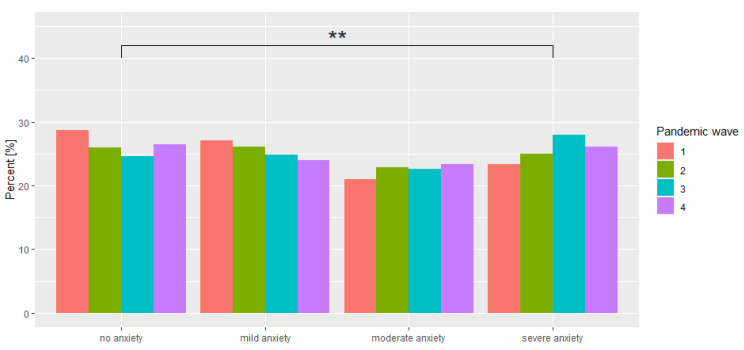
Comparison of GAD-7 scale interpretations across waves of the study. ** *p* < 0.01.

The situation is slightly different for the GAD-7 scale, which showed a significant increase only between waves 1 and 3 (*p* = 0.002) and waves 1 and 4 (*p* = 0.038). No significant differences were shown between the subsequent waves. Moreover, a slight decrease in mean scores was found between waves 3 and 4 (−0.0095; *p* = 0.995). When interpreting the GAD-7 scale score, it can be observed that between adjacent interpretations (no anxiety—mild anxiety (*p* = 0.999), mild anxiety—moderate anxiety (*p* = 0.783), moderate anxiety—severe anxiety (*p* = 0.999), as well as between no anxiety—moderate anxiety (*p* = 0.251), and mild anxiety—severe anxiety (*p* = 0.078) there is no statistically significant difference, while a statistically significant difference in the distribution of pandemic waves was noted for the levels of no anxiety—severe anxiety (*p* = 0.009). This is indicated by the fact that extreme GAD-7 scores changed significantly during the pandemic. A comparison is presented in Figure 2.

When analysing the MANSA scale, a statistically significant difference was found only for the second and third wave, where a decrease of 0.0846 points was observed (*p* = 0.013). An analysis of the individual questions comprising the MANSA scale shows that subjective satisfaction with one’s psychological well-being (*p* < 0.001) and extra-curricular activities (*p* < 0.001) decreased as the pandemic continued. A detailed comparative summary of the mean BDI, GAD-7 and MANSA scores is presented in Table 2.

### 3.3. Subjective Experience of Anxiety about Contracting COVID-19, and Being Quarantined or Infected by a Neighbour

**Figure 3:**
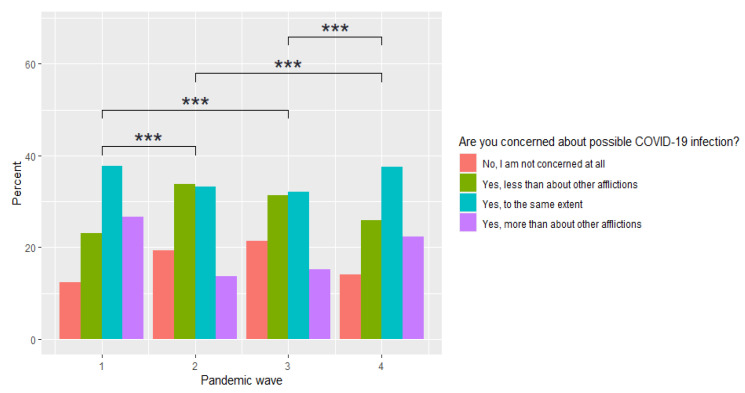
An assessment of changes in anxiety about COVID-19 compared to other conditions at different stages of the study. *** *p* < 0.001.

When analysing the question comparing the level of anxiety due to COVID-19 in relation to other conditions, a statistically significant difference of *p* < 0.001 was found, whereby, an increase in the percentage of people who were not afraid of the disease at all as the first, second and third waves of the pandemic continued, which then decreased at stage 4 observation shown in Figure 3.

The analysis of questions based on the Likert scale showed that between the first and third stages of the study there was a gradual decrease in the anxiety about one’s own disease, quarantine or neighbour’s disease. In stage 4 of the study, there was a change in the trend and each of the cases analysed showed an increase in value, and in the case of own disease this was the highest in the stage analysed. Similar observations were made in assessing adherence to government recommendations concerning combating the COVID-19 pandemic. A detailed comparison of mean scores is presented in Table 3.

### 3.4. The Influence of Sociodemographic Variables on the Mean Scores of BDI, GAD-7 and MANSA

A detailed summary of the influence of sociodemographic variables on the mean scores of BDI, GAD-7 and MANSA is presented in Table 4. The analysis of the entire study group indicated that the mean score of both BDI (*p* < 0.001) and GAD-7 (*p* < 0.002) decreased with increasing age. Similarly, men score significantly lower means than women on both scales. In terms of the level of education, it was shown that as the level of education increases, the mean scores on BDI and GAD-7 scales decrease and the quality-of-life score increases. Additionally, people in both marital and partnership relationships show better mental health and a higher quality of life. Undoubtedly, the reduced earning potential contributes significantly to increased levels of anxiety and depression and reduces the quality of life. People with pre-existing psychiatric conditions show higher BDI and GAD-7 scale values with concomitant lower quality of life scores.

### 3.5. The Relationship between GAD-7, BDI and MANSA Scales

A significant positive relationship between GAD-7 and BDI scales was observed at each stage of the study (Stage 1: r = 0.731, *p* < 0.001; Stage 2: 0.734, *p* < 0.001; Stage 3: r = 0.753, *p* < 0.001; Stage 4: r = 0.737, *p* < 0.001) and as BDI mean scores increased, so did the GAD-7. In contrast, a significant inverse relationship was found between both BDI and GAD-7 scales compared to MANSA (GAD-7 r = −0.573, *p* < 0.001; BDI r = −0.694, *p* < 0.001).

## 4. Discussion

Scientific reports to date indicate the negative impact of the COVID-19 pandemic on human mental health [8]. This study revealed significant differences in depressive and anxiety symptoms, as well as quality of life, between four waves of COVID-19 among Poles. It indicated that the most recent wave of infections had a less devastating impact on the mental health of respondents, which could confirm better mental adaptation with the duration of the pandemic or a better response to fewer restrictions than before. Several sociodemographic factors (such as age, sex, education, marital status) impacted substantially on the scores of the scales used.

The study was conducted in four stages for each incidence wave, and the results show a gradual increase in the frequency of depression and anxiety, as measured by BDI and GAD-7 scales. Nonetheless, this trend was not homogeneous and a slight reduction in depression and anxiety was observed in stage 4 of the study, which still exceeded the results observed in Poland before the pandemic [42]. According to studies on the Epidemiology of Mental Disorders and Access to Mental Health Care published in 2012, the prevalence of depressive disorders among Poles was 3%, and generalised anxiety disorder constituted 1.1% [43].

As already mentioned, the impact of the pandemic on psychological well-being was not homogeneous, and the greatest differences in anxiety and depression scores were shown between stages 1 and 3 of the observation. Firstly, this may be due to the significant disparity in infection and death rates between waves. During the first observations, the incidence rate was the lowest, ranging between 263 and 460 cases per day with a mortality rate of 18–40 per day, while during the third wave, the number of SARS-COV-2 infections was in the daily range of approx. 6–38,000, with the number of deaths between 428 and 954 per day [29]. Such extreme increases undoubtedly had a negative impact on the sense of security concerning the life and health of oneself and one’s loved ones, which worsened psychological well-being [44]. Significant increases may also result from chronic fatigue in society with long-term restrictions and limitations in daily functioning in response to ongoing restrictions [30,31,32,33]. Observations from, e.g., England and Hong Kong stress the negative impact of lockdown on the mental health of citizens, which seems to prove the present hypothesis [45,46]. In contrast, the liberal approach to restrictions during the fourth wave of the pandemic may have affected the relative improvement in the mental health of citizens, notwithstanding the highest morbidity and mortality rates among COVID-19 patients [29]. During the fourth wave of the pandemic, shopping centres and shops remained open, and 75% of seats were made available for use at sports venues, cinemas and restaurants [33]. This fact made it possible to increase the mobility and frequency of people-to-people contacts. Geolocation data from mobile phones revealed that mobility and, thus, social and cultural activities of the Polish people were not potentially reduced, and such a phenomenon was evident for each of the previous three waves of the pandemic [47]. Physical activity contributes to improved mood and cognitive function. The opening of gyms enabled people to take care of themselves in the former way [48]. This lifestyle may have provided a substitute for a relative return to pre-pandemic conditions. This may also be evidenced by the increase in responders’ subjective ratings of physical fitness and satisfaction with their hobbies between waves 3 and 4 of the pandemic. A similar relationship was reported in a study from Austria, where there was a reduction in anxiety after the withdrawal of numerous coronavirus restrictions [49]. The results of this study are also consistent with reports from the United Kingdom and China, where the highest levels of fear and anxiety were found during the peak of the pandemic and then they decreased during the period of loosening of coronavirus restrictions [50,51]. The reduction in the incidence of depression and anxiety in the last stage of the study may also be due to human adaptability to the new reality. Previous findings have confirmed that the process of adapting to new conditions is long but inevitable [52]. It requires the development of appropriate mechanisms to adapt and cope with stress [52]. There was a similar decreasing trend in the severity of fear and anxiety during the influenza A virus subtype H1N1 pandemic in the Netherlands [53]. Similar findings were reported in the USA and the Netherlands where, despite an increase in SARS-COV-2 cases, there was no relevant increase in depressive or anxiety symptoms [49,54,55].

There is a noteworthy trend change in the fourth pandemic wave for the subjective concern about becoming infected with COVID-19 or infecting family and friends, as well as for adherence to coronavirus restrictions. It might have been a result of the relatively low average age of responders, who may have felt more threatened in this wave because the Delta variant was more threatening for this age group than previous variants [5,6,7,8]. Interestingly, in another study, the Polish people were much more afraid of COVID-19 infection compared to Israelis or Canadians, which was associated i.e., with the difference in terms of the quality of medical services in these countries [56].

Another analysed factor was quality of life that, according to the WHO’s definition, is a subjective assessment of one’s own life situation with respect to many aspects of life. This measurement is important due to its ability to assess a patient’s perspective of an ongoing situation. When assessing the quality of life, it is necessary to bear in mind that it is a complex parameter that can be influenced by many variables. Our observation initially revealed a decrease in quality of life in the first three waves of the COVID-19 pandemic. There was a slight increase in the last stage of the study. However, the analysis of individual questions of the MANSA scale revealed that during the final stage of the survey, responders rated subjective life satisfaction, relationship with family or housing situation significantly higher compared to the previous pandemic wave. On the other hand, there was a decline in the sense of security and the quality of friendships. It should also be noted that the deteriorating economic situation of citizens contributes to the exacerbation of negative emotions and a decrease in quality of life [49]. A population-based study conducted in Japan revealed a statistically significant reduction in quality of life one year after the pandemic outbreak. Likewise, in this study, socioeconomic parameters had a major impact on the responders’ scores [57]. The implementation of the lockdown has had a considerable impact on the economy, resulting in many people working reduced hours or losing their jobs altogether [44]. Soaring inflation caused by the pandemic has contributed greatly to the decline in life satisfaction [58]. Importantly, a stable financial situation provides a sense of security and a belief that in the event of illness there will be funds to obtain treatment, which reduces fear/anxiety [16]. Corresponding results were obtained in previous studies conducted among the Chinese population [1]. Another factor that could affect the quality of life was the possibility of the use of immunisation. According to research reports, fully vaccinated individuals had considerably higher MANSA scores compared to unvaccinated individuals [22].

The results of this study indicate that women and individuals with a psychiatric history are more prone to develop depressive and anxiety disorders. Data from the 2014 European Health Interview Survey also found a considerable difference in terms of well-being between men and women. In that study, 8.8% of women reported a history of chronic depression, compared to only 5.3% of men who struggled with the illness [42]. On the one hand, these differences may be due to the individual predisposition of women to develop mental disorders. On the other hand, however, these differences may also be due to the greater propensity of women to report problems [42]. Another factor that affects the greater escalation of symptoms in women may have been the closure of kindergartens and nurseries, which resulted in shifting more responsibility for child learning and supervision to mothers [59].

This study has also proved that people with mental illnesses score higher on both BDI and GAD-7 scales. This may be because severe mental illnesses such as schizophrenia or bipolar disorder are associated with a higher risk of severe COVID-19 course or COVID-19-related death [60,61]. Moreover, the overloading of the health care system during the pandemic resulted in reduced access to psychiatrists, which may have resulted in an exacerbation of pre-existing psychopathological symptoms [62]. Convergent observations were found in the world literature. A study conducted on the German population also proved the greater vulnerability of previously psychiatrically treated individuals to depression and anxiety escalation during the COVID-19 pandemic [63].

According to this study, younger people scored higher on the GAD-7 and Beck’s Depression Inventory, which is consistent with observations from Australia that suggest an effect of the introduction of distance learning on anxiety escalation [64]. Similar observations were found regarding remote work [65]. Moreover, reduced autonomy, loneliness and difficulties in relationships with parents contributed to increased negative emotions [16,64]. Younger people reveal a greater preference for quantity over quality of social interactions, which aggravates the adverse effects of loneliness during the pandemic and lockdown [66]. Moreover, quarantines caused the frequent separation of family members, and young people are, again, most affected by the psychological harm resulting from it [67].

The authors are aware of the limitations of this study, one of which is undoubtedly the lack of representativeness of the study group with respect to Polish society. The predominance of women and the average age of the responders may have biased the final results of this study due to the greater vulnerability of the aforementioned groups to the escalation of depressive and anxiety symptoms [45]. Moreover, the number of respondents in each stage of the study is unequal. Another limitation is the method of data collection through an anonymous survey that was distributed through social networking sites. However, the survey was shared in groups with a diverse range of topics to reduce convenience sampling. Therefore, only those with access to the Internet could participate in the survey, and the authors of this study were unable to verify the personal details of the participants. On the other hand, due to the current sanitary and epidemiological situation, online surveys are a safe form of research and provide an opportunity to reach a large number of respondents. It should also be noted that those concerned about their health may have been more interested in participating in the study.

Further research based on longitudinal methodology is needed to study the impact of the pandemic on the mental and physical health of citizens. Its results will provide valuable guidance to clinicians, mental health nurses and psychologists, and will enable the prevention of acute and chronic mental disorders in the future [68].

## 5. Conclusions

The first three waves of the pandemic were characterised by a deterioration of mental health in the form of anxiety and depression. Factors that increase anxiety and depression include younger age, female sex and reduced earning capacity. The difference in the results for the fourth wave of the pandemic may result from the respondent’s adaption to the situation and less severe restrictions. There is a close relationship between the severity of anxiety/depression and the subjective assessment of the quality of life. There is a need to continue monitoring human mental health during the COVID-19 pandemic.

## Figures and Tables

**Table 1:** Characteristics of the study group by different stages of the study.

Variable		Stage 1 (*n* = 2467) M ± SD/N (%)	Stage 2 (*n* = 1627) M ± SD/N (%)	Stage 3 (*n* = 1696) M ± SD/N (%)	Stage 4 (*n* = 949) M ± SD/N (%)	*p*
Age		32.2 ± 10.72	24.6 ± 7.06	27.83 ± 9.55	29.7 ± 9.60	<0.001
Sex	Female	2037 (82.5)	1295 (79.6)	1394 (82.2)	789 (83.1)	0.003
	Male	430 (17.5)	332 (20.4)	302 (17.8)	160 (16.9)	
Place of residence	Rural area	461 (18.7)	287 (17.6)	326 (19.2)	184 (19.4)	0.002
	Town of up to 50,000 inhabitants	377 (15.3)	233 (14.4)	268 (15.8)	136 (14.3)	
	City of 50,000–250,000 inhabitants	449 (18.2)	303 (18.6)	353 (20.8)	215 (22.7)	
	City of over 250,000 inhabitants	1180 (47.8)	804 (49.4)	744 (44.2)	414 (43.6)	
Level of education	Higher (university degree)	1481 (60.0)	513 (31.5)	654 (38.6)	493 (52.0)	<0.001
	Incomplete higher	514 (20.8)	646 (39.6)	543 (32.1)	222 (23.4)	
	Secondary	429 (17.4)	437 (26.9)	445 (26.4)	227 (23.9)	
	Vocational	26 (1.0)	8 (0.5)	9 (0.5)	4 (0.4)	
	Lower secondary	13 (0.6)	19 (1.2)	24 (1.4)	2 (0.2)	
	Primary	4 (0.2)	4 (0.3)	9 (0.5)	1 (0.1)	
Marital status	Married	867 (35.1)	163 (10.0)	323 (19.0)	352 (37.1)	<0.001
	Partnership	556 (22.6)	446 (27.5)	475 (28.0)	226 (23.8)	
	Widowed	30 (1.2)	7 (0.4)	14 (0.8)	3 (0.3)	
	Divorced	108 (4.4)	25 (1.5)	50 (3.0)	21 (2.2)	
	Single	905 (36.7)	986 (60.6)	834 (49.2)	347 (36.6)	
Healthcare professional	Yes	632 (25.6)	203 (12.5)	245 (14.5)	163 (17.2)	<0.001
Prior psychiatric treatment	Yes	516 (20.9)	333 (20.5)	340 (20.1)	168 (17.7)	0.209
Psychiatric medication treatment	Yes	443 (18.0)	268 (16.5)	283 (16.7)	157 (16.5)	0.543
Recent Suspicion of COVID-19	Yes	78 (3.2)	323 (19.9)	352 (20.8)	162 (17.1)	<0.001
Compulsory quarantine in the current wave	Yes, I am under quarantine	23 (0.9)	29 (1.8)	31 (1.8)	22 (2.3)	<0.001
	Yes, I was under quarantine	59 (2.4)	243 (14.9)	314 (18.5)	125 (13.2)	
COVID-19 diagnosis	In the course of the disease	189 (7.9)	33 (2.0)	39 (2.3)	18 (1.9)	<0.001
	Yes, I was infected with COVID-19 in the past	143 (6.0)	248 (15.2)	298 (17.6)	96 (10.1)	
COVID-19 diagnosed in closest relatives	Yes	117 (4.7)	1036 (63.7)	1122 (66.2)	452 (47.6)	<0.001
Information retrieval	Yes	1530 (62.0)	776 (47.7)	767 (45.22)	547 (57.6)	<0.001
Tracking statistics on COVID-19	Yes	1562 (63.3)	781 (48.0)	710 (41.9)	478 (50.4)	<0.001
Loss of income opportunities	Yes	610 (24.7)	340 (20.9)	359 (21.2)	124 (13.1)	<0.001

**Table 2:** Comparison of BDI, GAD-7 and MANSA scores according to different stages of the study.

Beck’s Depression Inventory (BDI)					
Wave	Wave	Difference in Means	Lower End of the Range for Differences	Upper End of the Range for Differences	*p* #
1	2	0.0972	0.0583	0.136	<0.001
1	3	0.138	0.0984	0.178	<0.001
1	4	0.0806	0.0325	0.129	<0.001
2	3	0.0413	−0.00367	0.0862	0.085
2	4	−0.0166	−0.0689	0.0357	0.848
3	4	−0.0578	−0.111	−0.00465	0.027
GAD-7					
1	2	0.0560	−0.0155	0.127	0.183
1	3	0.101	0.0286	0.173	0.002
1	4	0.0916	0.00335	0.180	0.038
2	3	0.0447	−0.0335	0.123	0.456
2	4	0.0357	−0.0577	0.129	0.759
3	4	−0.00905	−0.103	0.0849	0.995
MANSA					
1	2	0.0575	−0.00747	0.122	0.104
1	3	−0.0271	−0.0927	0.0384	0.711
1	4	0.0444	−0.0347	0.124	0.473
2	3	−0.0846	−0.156	−0.0131	0.013
2	4	−0.0131	−0.0973	0.0711	0.979
3	4	0.0716	−0.0131	0.156	0.131

# (Welch’s) ANOVA univariate.

**Table 3:** The comparison of the mean values of the level of anxiety of COVID-19, anxiety due to quarantine or neighbour’s disease, and the degree of adherence to the government’s COVID-19 pandemic control recommendations for each wave of the COVID-19 pandemic.

	Wave 1	Wave 2	Wave 3	Wave 4	*p* *
Anxiety about being infected with COVID-19 disease					
Mean	5.51	4.86	4.92	5.77	<0.0001
Comparison of individual COVID-19 pandemic waves			x	x	<0.0001
		x		x	<0.0001
		x	x		0.0372
	x			x	0.9996
	x		x		<0.0001
	x	x			<0.0001
Anxiety about neighbours being infected with COVID-19					
Mean	5.73	3.63	3.59	3.91	<0.0001
Comparison of individual COVID-19 pandemic waves			x	x	<0.0001
		x		x	<0.0001
		x	x		<0.0001
	x			x	0.3327
	x		x		0.1562
	x	x			0.0009
Anxiety about neighbours in quarantine					
Mean	4.64	3.03	2.93	3.23	<0.0001
Comparison of individual COVID-19 pandemic waves			x	x	<0.0001
		x		x	<0.0001
		x	x		<0.0001
	x			x	0.4537
	x		x		0.7136
	x	x			0.0371
Adherence to the Ministry of Health recommendations regarding COVID-19 prevention					
Mean	8.67	7.63	7.10	7.49	<0.0001
Comparison of individual COVID-19 pandemic waves			x	x	<0.0001
		x		x	<0.0001
		x	x		<0.0001
	x			x	<0.0001
	x		x		0.9641
	x	x			<0.0001

* Type-II ANOVA.

**Table 4:** Effects of sociodemographic variables on scores of individual scales.

		BDI				GAD-7				MANSA			
		Value	SD	t	*p*	Value	SD	t	*p*	Value	SD	t	*p*
Age		−0.097	0.019	−5.15	**0.0000**	−0.035	0.011	−3.10	**0.0020**	−0.002	0.024	−0.11	0.9145
Sex	Male	−1.601	0.323	−4.96	**0.0000**	−2.020	0.194	−10.38	**0.0000**	−0.218	0.406	−0.54	0.5917
Place of residence	Rural area	0.230	0.340	0.68	0.4986	0.084	0.206	0.41	0.6817	−0.184	0.427	−0.43	0.6656
	Town of up to 50,000 inhabitants	0.157	0.368	0.43	0.6699	−0.020	0.223	−0.09	0.9268	−0.409	0.463	−0.88	0.3764
	City of 50,000–250,000 inhabitants	−0.118	0.335	−0.35	0.7236	−0.120	0.203	−0.59	0.5547	0.339	0.420	0.81	0.4204
Level of education	Higher (university degree)	−9.487	2.798	−3.39	**0.0007**	−3.544	0.826	−4.29	**0.0000**	8.701	1.707	5.10	**0.0000**
	Incomplete higher	−7.110	2.821	−2.52	**0.0118**	−2.880	0.829	−3.47	**0.0005**	6.480	1.713	3.78	**0.0002**
	Secondary	−5.925	2.828	−2.09	**0.0362**	−2.702	0.832	−3.25	**0.0012**	5.758	1.719	3.35	**0.0008**
	Vocational	−8.500	3.412	−2.49	**0.0128**	−1.288	1.146	−1.12	0.2612	2.692	2.367	1.14	0.2554
	Primary	6.250	5.7446	1.09	0.2766	−2.900	1.634	−1.77	0.0760	8.115	3.375	2.40	**0.0162**
Marital status	Married	−2.824	0.48	−5.88	**0.0000**	−0.384	1.723	−0.22	0.8235	2.950	0.606	4.87	**0.0000**
	Partnership	−1.392	0.544	−2.56	**0.0106**	−0.168	0.332	−0.51	0.6120	2.869	0.684	4.19	**0.0000**
Medic	Yes	−1.939	0.467	−4.14	**0.0000**	−0.037	0.285	−0.13	0.8949	2.356	0.406	5.81	**0.0000**
Earnings reduction	Yes	3.249	0.467	6.95	**0.0000**	1.267	0.286	4.43	**0.0000**	−4.757	0.587	−8.10	**0.0000**
Psychiatric treatment	Yes	5.681	0.302	18.80	**0.0000**	2.945	0.301	9.79	**0.0000**	−6.364	0.624	−10.2	**0.0000**
Vaccinations against COVID-19	No	−0.897	0.212	4.23	**0.00001**	0.038	0.123	0.31	**0.755**	−0.727	9.255	−2.85	**0.004**

Statistically significant values are in bold with the significance level set at *p* < 0.05.

## Data Availability

The data presented in this study are available on request from the corresponding author.
